# The Relationship between Concurrent Substance Use Disorders and Eating Disorders with Personality Disorders

**DOI:** 10.3390/ijerph6072076

**Published:** 2009-07-23

**Authors:** Christine Courbasson, Jacqueline M. Brunshaw

**Affiliations:** 1 Department of Psychiatry, University of Toronto, Centre for Addiction and Mental Health, 33 Russell Street, Toronto, Ontario, M5S 2S1 Canada; 2 Eating Disorders and Addiction Clinic, Concurrent Disorders Service, Centre for Addiction and Mental Health, 33 Russell Street, Toronto, Ontario, M5S 2S1 Canada; E-Mail: JBrunshaw@mindandsoul.ca

**Keywords:** substance use disorders, eating disorders, personality disorders, avoidant personality disorder, borderline personality disorder, obsessive compulsive personality disorder

## Abstract

**Objective:**

The current pilot study investigated whether patients with concurrent substance use disorders and eating disorders (SUD and ED) who experienced a reduction in SUD and ED symptoms following treatment for SUD and ED also experienced a reduction in personality disorder (PD) symptoms.

**Method:**

Twenty patients with SUD and ED and PD were assessed pre and post treatment using clinical interviews, self-report questionnaires, and a therapist questionnaire on DSM-IV-TR symptoms for PD.

**Results:**

Symptoms for the personality disorders were reduced following treatment. This reduction was correlated with a decrease in the number of symptoms of ED at post treatment.

**Discussion:**

Chronic concurrent SUD and ED may make it difficult to separate PD symptoms from co-occurring disorders. Many features attributed to PDs may be reduced when problematic substance use and disordered eating are addressed, a fact that may increase clinician and patients’optimism about therapeutic change.

## 1. Introduction

A growing body of literature suggests that there is substantial comorbidity between substance use disorders (SUD) and eating disorders (ED). Depending on the criteria used to make the diagnoses, ED can occur in up to 32% of substance use cases [1–9]. In turn, substance use has been found to occur in up to 49% of people with ED [10–19].

Individuals with concurrent SUD and ED are frequently diagnosed with multiple comorbid clinical disorders, including personality disorders (PDs) (between 11.7–26%) [20–22]. Personality disorders are characterized by “an enduring pattern of inner experience and behavior that deviates markedly from the expectations of the individual’s culture, is pervasive and inflexible, has an onset in adolescence or early adulthood, is stable over time, and leads to distress or impairment” [23]. In the general community, the most prevalent PDs are Borderline Personality Disorder (BPD), affecting 2% of adults, mostly young women [24], Avoidant Personality Disorder (Avoidant), affecting 2.4% of adults, and Obsessive-Compulsive Personality Disorder (OCPD) affecting 7.9% of adults [25]. BPD is defined as “a pervasive pattern of instability of interpersonal relationships, self-image, and affects, and marked impulsivity” [26]. Avoidant PD is defined as “a pervasive pattern of social inhibition, feelings of inadequacy, and hypersensitivity to negative evaluation” [27]. OCPD is defined as “a pre-occupation with orderliness, perfectionism, and mental and interpersonal control” [28].

Comorbidity of the Cluster B PD has been found to be more common for patients with concurrent SUD and ED, than for those with ED only [29]. In patients diagnosed with anorexia nervosa (AN) and bulimia nervosa (BN), 56% have also been diagnosed with comorbid PDs [30]. In patients diagnosed with binge-eating disorder (BED), 20% have been found to meet criteria for concurrent PDs [31]. Additionally, up to 65% of individuals seeking treatment for SUD have been diagnosed with active BPD [32]. In a sample of treatment-seeking patients diagnosed with concurrent SUD ED, 26% were diagnosed with comorbid BPD, 18.2% with OCPD, and 11.7% with Avoidant PD [33]. This comorbidity carries a high individual and societal cost. These individuals have difficulty maintaining employment, financial stability, and meaningful interpersonal relationships [30,31].

Clinicians involved in the treatment of concurrent SUD and ED at an Eating Disorders and Addiction Clinic (EDAC) at a large mental health and research facility have reported for several years that most individuals who meet criteria for PDs at the start of treatment no longer meet the criteria after treatment for concurrent SUD and ED. A few published studies provide support for these clinical observations. In a sample of patients with long standing eating disorders, Oyvind and colleagues [34] reported a high commorbidity of personality disorders and a reduction in PDs from 77% at admission to an eating disorder treatment to 57% at 2-year follow-up. In a 5 year follow-up study, Vrabel, Martinsen, Hoffart and Rosenvinge [35] found that patients who recovered from ED had a lower frequency of PDs. Oyvind and colleagues [36] observed that in general, ED symptomatic changes occurred prior to changes in PD, thus suggesting that PD could be a consequence of ED symptomatology. While a reduction of PD has been observed following treatment for ED, it is not known if similar findings can occur in a sample of clients with concurrent ED and SUD as SUD is associated with poor treatment outcome [37].

The objective of this pilot study was to assess whether patients with concurrent SUD, ED, and PDs experienced a reduction in the number of PD symptoms after receiving treatment for their concurrent SUD and ED. A decrease in the number of symptoms and diagnoses was explored specifically for the three most frequently occurring PDs in the concurrent SUD and ED population presenting for concurrent treatment, and reported in the literature [38,39]. These three PDs are Borderline, Avoidant, and Obsessive-Compulsive.

It was hypothesized that patients who participated in a concurrent treatment program for SUD and ED and who experienced a significant reduction of SUD and ED symptoms would be more likely to significantly reduce their BPD, Avoidant PD, and OCPD symptoms following treatment compared to those patients who were less successfully treated for their concurrent SUD and ED.

## 2. Method

### 2.1. Participants and Procedure

The sample consisted of 20 outpatients who completed a concurrent treatment program for SUD and ED within a large metropolitan mental health centre. Exclusion criteria for the study included patients demonstrating evidence of mental retardation, active psychosis, or chronic self-harm and/or suicidal attempts/gestures. The sample was comprised of 17 (85%) females and three (15%) males diagnosed at pre-treatment with active SUD, ED and PDs (BPD, Avoidant PD, and OCPD).

Patients were administered the Structured Clinical Interview for DSM (SCID) Axis I diagnoses [37], for Axis II diagnoses [38], and other SUD, ED, and mental health assessment measures (see Section 2.2.1.) to determine eligibility in the treatment and severity of symptoms. Subsequently, they were asked to volunteer for a study on concurrent SUD and ED. All patients who were informed about the study agreed to participate, Patients eligible for enrollment in a concurrent treatment program for SUD and ED were free to accept, reject, and withdraw from the treatment and study at any time. All patients provided informed signed consent for their data to be used for research purposes, and ethical guidelines were strictly followed. Patients received an outpatient psychobehavioural specialized treatment for concurrent SUD and ED that included weekly individual and group treatment, as well as meetings with dieticians and psychiatrists as needed. The individual and group psychotherapy treatment included psychoeducation about SUD, ED, coping and nutrition. Motivational interviewing, mindfulness training, and BT/CBT/DBT were interventions employed. The focus of the treatment was to eliminate or reduce substance use and eating disordered behaviours. It was not to target PD. Patients monitored their substance use and ED behaviours and urges in daily logs and discussed these in groups as well as problems they may have had implementing adaptive coping strategies. Patients learned adaptive techniques to reduce their urges to use substances or engage in problematic eating and restricting. They were taught to identify personal triggers and reduce the vulnerability to those triggers. They learned to adopt healthier eating and sleeping habits and ways to monitor their emotions and respond adaptively (as opposed to react negatively) to them. Patients were guided on living in the moment as opposed to dwelling on the past and/or worrying about the future. They were also trained to develop and practice assertiveness skills, create a balanced lifestyle, identify and challenge dysfunctional cognitions, explore new activities and interests, eliminate toxic relationships and develop new healthier ones.

There was variability in the amount of treatment received, but on average patients attended 67.8 (SD = 46) individual sessions, 49.1 (SD = 29.7) 2 hour group sessions, 2.5 (SD = 4.7) appointments with a dietician, and 11.4 (SD = 9.2) phone coaching sessions. In addition, six patients also attended an average of 7.7 (SD = 5.5) appointments with a psychiatrist within the program (to prescribe and monitor the use of psychotropic medication). Six of the patients were prescribed psychotropic medication for at least a portion of their treatment. These medications included Serzone, Effexor, Celexa, Fluoxetine, Prozac, Diazepam, and Surmontil. Two patients attended appointments with a counselor (four and 28 sessions) to deal with addiction medicine issues (one case involving methadone treatment), and one patient received 15 vocational counseling sessions. Patients remained in the program from 12 to 45 months, with an average of 21.4 months (SD = 10.2).

Assessments of patients were completed at pre-treatment and post-treatment. Clinicians involved in the assessments and treatments were blind to the study hypotheses. A doctoral-level research associate who was not involved in the assessment or treatment of these patients conducted the review of data for all patients who had completed the treatment program. To explore whether a reduction in the number of symptoms of SUD, ED, and the three PDs of interest (BPD, Avoidant PD, and OCPD) occurred at post-treatment, data was collected for both the pre- and post-treatment assessment phases.

### 2.2. Sources of Data

The following measures have been chosen for their excellent psychometric properties and ease of administration.

***Structured Clinical Interview for DSM diagnoses (SCID)*** [40,41]. The SCID is a structured interview administered by trained clinicians to determine presence or absence of DSM Axis I clinical diagnoses and Axis II personality disorders. It is one of the most frequently used instruments to identify clinical and personality disorders in clinical research. In this study, the SCID was used to determine treatment eligibility and confirm presence of active SUD, ED, and the three PDs of interest. Presence of other concurrent Axis I and II disorders were also identified. In addition to the SCID, symptoms of SUD, PD and ED were assessed with the following respective instruments.

***Beck Depression Inventory*** (BDI) [42]. The BDI is a 21-item self-report questionnaire which measures symptoms of depressed mood, including suicidal ideation, irritability, and anhedonia. Current severity of symptoms over the past week is assessed. Higher scores reflect greater severity of depression.

***Beck Anxiety Inventory*** (BAI) [43]. The BAI is a 21-item self-report questionnaire which measures general symptoms of anxiety, including physiological responses. Current severity of anxiety over the past week is assessed, and higher scores reflect a greater level of anxiety.

***Symptom Checklist-90-Revised*** (SCL-90-R) [44]. The SCL-90-R is a 90-item self-report symptom inventory that assesses psychopathology and measures current level of distress (over the past two weeks) on nine primary symptom dimensions and three global indices of distress. The dimensions include: Depression, Anxiety, Paranoid Ideation, Psychoticism, Interpersonal Sensitivity, and Obsessive-Compulsive. Higher scores reflect greater level of distress and psychological disorder.

***Reflective Activity Scale*** (RAS) [45]. The RAS is a 39-statement inventory that assesses individual differences along a continuum ranging from being excessively preoccupied and inactive, to reflectively active, to thoughtlessly reactive or impulsive. The RA scale assesses the four dimensions of: (1) reflection, (2) action, (3) information gathering, and (4) over-absorption, with regards to facing problematic situations. Higher scores reflect higher tendency to reflect, take action, gather information, and be overly absorbed in the face of stressful situations.

***Toronto Alexithymia Scale-20*** (TAS-20) [46]. The TAS is a 20-item scale that assesses alexithymia or affective deficits on three factors: Difficulty in Identifying Feelings, Difficulty in Describing Feelings, and Externally-Oriented Thinking. A total score is also calculated. Scores range from 20–100. Scores of 61 or higher indicate alexithymia.

***The Eating Disorder Inventory*** (EDI) [47]. The EDI is a 64-item self-report questionnaire which measures attitudes, feelings, and behaviours related to eight dimensions of eating disturbance: Bulimia, Perfectionism, Drive for Thinness, Interoceptive Awareness, Interpersonal Distrust, Ineffectiveness, Body Dissatisfaction, and Maturity Fears. Higher scores reflect greater disturbance on the eating dimensions.

***Eating Disorders Examination*** (EDE) [48]. The EDE is a semi-structured interview which assesses rigidity and severity of symptoms on four attitudinal disturbances related to eating disorders: Restraint, Eating Concern, Weight Concern, and Shape Concern. A Global Index of Severity of symptoms is also determined. Higher scores reflect greater severity in eating disturbance.

***Addiction Severity Index*** (ASI) [49]. The ASI is a structured interview which evaluates addiction severity and psychosocial impairment in six areas: 1) alcohol and drug abuse (e.g., number of overdoses, money spent on substances); 2) medical (e.g., hospitalizations); 3) employment (e.g., net income); 4) legal (e.g., number of convictions); 5) family/social (e.g., relationship discord); and 6) psychiatric problems (e.g., suicidality). Questions include the number, duration, and extent of problems in the patient’s lifetime and in the past 30 days. Higher scores reflect greater severity in addiction.

***Drug-Taking Confidence Questionnaire for Alcohol*** (DTCQ-A) ***and for Drugs*** *(DTCQ-D)* [50]. The DTCQ-A and DTCQ-D are the 8-item brief versions of the original 50-item self-report questionnaires. These questionnaires measure impulsivity, coping self-efficacy, and level of confidence in being able to resist using a substance across different categories of high-risk-for-relapse crisis situations. Lower scores reflect lower confidence, lower coping self-efficacy, and greater impulsivity.

***Therapist Questionnaire*** (TQ) [51]. The TQ designed for the study was used to identify PD psychopathology relating to BPD, Avoidant PD, and OCPD. The TQ contains 63 questions relating to all of the clinical features corresponding to BPD, Avoidant PD, and OCPD listed in the DSM-IV [52]. Therapists were asked to indicate presence of each symptom at pre-therapy and the presence, absence, or improvement of each symptom at post-therapy. Ratings were made at both pre-and post-treatment.

### 2.3. Statistical Analysis

Information from the standardized measures and therapist questionnaire were included in the data analysis to provide an indication of convergent validity to the responses by patients on their self-report measures. Change in symptoms of SUD, ED, and PD was computed by subtracting pre-treatment scores on the measures from post-treatment scores. Paired t-tests and chi-squared tests (as appropriate) were conducted to assess change from pre- to post-treatment. Pearson correlations were conducted to identify association amongst relevant variables. Significance levels were corrected using Bonferroni adjustment for multiple comparisons.

## 3. Results and Discussion

The age of the patients in the study ranged from 21 to 59 years (mean = 36.6, SD = 2.04). Over a third of patients (35%) were single/never married. Twenty-five percent of patients had completed university, and 20% had completed some university. Most patients (60%) were either unemployed or on disability at the start of treatment and 5% were employed part-time. Most patients (60%) reported alcohol as their primary substance of concern. Half (50%) of the patients were diagnosed with BN at pre-treatment, and 30% with AN (Binge-Purge sub-type). As well, 40% of patients were diagnosed with BPD, 35% with Avoidant PD, and 30% with OCPD (these PD diagnoses were not mutually exclusive). One-quarter (25%) of patients had had previous therapy for an ED, and most patients (65%) were taking prescribed anti-depressants at pre-treatment.

The number of personality symptoms present at pre-treatment for each of the three PDs (as indicated on the TQ) were statistically significantly reduced at post-treatment for BPD (χ^2^ = 39.744, df = 24, t = 2.216, p = 0.040); for Avoidant (χ^2^ = 43.214, df = 18, t = 2.717, p = 0.014); and for OCPD (χ^2^ = 22.361, df = 24, t = 2.396, p = 0.028). Such statistically significant reductions in personality disorder symptoms are clinically significant as well given that in most cases patients’ diagnoses remitted during treatment. All nine patients who initially met criteria for OCPD, all but one of twelve patients with BPD, and all but one of twelve patients with APD, no longer met the diagnoses following treatment. The number of PD symptoms as measured by the TQ also decreased substantially from a mean of 4.6 (SD = 2.67) BPD symptoms to 1 (SD = 2.2.), from a mean of 2.7 OCPD symptoms (SD = 2.25) to 0.4 (SD = 0.821), and from a mean of 3.95 (SD = 2.7) to 0.4 (SD = 0.35) post treatment.

As seen in Table 1, the results of paired t-test calculations showed significant decreases in the number of symptoms from pre- to post-treatment on various indices that measured relevant PD symptoms (see Table 1), and on the various indices of SUD and ED (see Table 2). For many of these scales and variables, mean scores changed from a greater category of severity to a lower category of severity. For example, the Global Symptom Index of the SCL-90 changed from a mean T score of 69.78 (almost the 98th percentile), to 58.78, which is within the normal range (below one SD above the mean). This is the case for five out of the six subscales (the exception being Obsessive Compulsive) on this measure as well. For the BDI, patients’ mean score changed from being in the moderate severe range to the minimal range, and for the BAI, patients’ mean score changed from the moderate severe range to mild range. Patients’ mean scores were in the average range post- treatment for the following various scales that were elevated prior to treatment: Alexithymia, Interpersonal Distrust, Bulimia, Global Index of the Eating Disorder Examination, Drive for thinness, and Interceptive awareness.

The PD symptom reductions were statistically significantly correlated with a decrease in number of symptoms of ED symptoms (on the EDE) at post-treatment for Avoidant PD (r = 0.686, p = 0.010), but not for OCDP (r = 0.311, p = 0.300) and BPD (r = 0.446, p = 0.146). Although there were no statistically significant correlations between PD symptom reductions and post-treatment decrease in SUD symptoms, a trend towards a decrease was apparent for BPD. The non-significance may be more the result of lack of power than lack of effect (r = 0.409, p = 0.211 for BPD, r = −0.118, p = 0.731 for Avoidant PD, r = 0.146, p = 0.668 for OCPD).

## 4. Conclusions

This pilot study explored whether patients with concurrent SUD, ED, and PDs experienced a reduction in the number of PD symptoms after receiving treatment for their concurrent SUD and ED. The PDs of interest were BPD, Avoidant PD, and OCPD. Half of the patients were diagnosed with BN and a third with AN (Binge-Purge sub-type). Almost half were diagnosed with BPD, over a third with Avoidant PD, and almost a third with OCPD. Their primary substance use of concern was alcohol. After undergoing concurrent SUD and ED treatment for an average length of almost 2 years, patients showed a reduction of SUD, ED, and PD symptoms that were both statistically and clinically significant. These results are consistent with research on patients with ED who have similarly experienced a decrease in their ED and PD symptoms over the course of treatment for their ED [34].

In our study, the PD symptom reductions were associated with a decrease in number of ED symptoms at post-treatment particularly evident for Avoidant PD. There was a trend for ED and SUD symptom reduction to be associated with a reduction in BPD symptoms. A larger sample size may clarify these findings. Although this is a pilot study and the number of patients is too low to draw firm definite conclusions about the relationship between concurrent SUD/ED and PD, the results raise several hypotheses to consider. First, the treatment may have affected the SUD, ED, and PD symptoms simultaneously. Treatment designed to decrease problematic substance use and eating often involves changing how a person manages emotions, copes with stress, resolves problems, relates to others, and thinks about him/herself and the world, all of which can be challenges for people with BPD, APD or OCPD. Working on such goals may inadvertently decrease PD symptomatology.

Second, the reduction in ED and SUD symptoms may have resulted in a reduction of PD symptoms. Efforts to maintain an ED and SUD may require significant behavioural and personality changes, such as avoidance of people (a symptom of Avoidant PD) due to shame about disordered behaviour, disgust about appearance, or to prevent interruption during problematic eating and substance use. Similarly, patients with eating disorders may develop rigidity and compulsivity in adhering to strict routines to ensure that they do not gain weight, symptoms associated with OCPD. Additionally, patients with ED and SUD symptoms may develop PD like symptoms because of the physiological effects of their ED and SUD behaviours, such as difficulties in concentration and thinking, paranoid thinking, affective instability, all of which are common in BPD. When the ED and SUDs are in remission, these developed behaviours and personality changes may no longer be necessary and will begin to subside. Structural equation modeling used by Oyvind and colleagues [34] supports this hypothesis. In their hospital sample, patients exhibited changes in their eating disorder symptoms prior to changes in their personality disorder symptoms.

Third, the reduction in PD symptoms might actually precede the reduction in ED and SUD symptoms, indicating that treatment works to reduce problematic personality and behavioural tendencies that maintain ED and SUD symptoms. The treatment provided within this study was lengthy, comprehensive, and intense, and may have served to first reduce PD symptoms, which later may have reduced the need to engage in problematic eating and substance use.

Finally, the reduction in ED, SUD, and PD symptoms might reflect a problem with diagnostic assessments. Many symptoms that are characteristic of PDs may be attributable to SUD and ED. For example, one of the symptoms of BPD is impulsivity in areas that are self-damaging, such as substance use or binge eating, which of course are present in ED and SUD. Other symptoms that are often present in ED, SUD, and PD include interpersonal sensitivity, interpersonal distrust, perfectionism, fear of criticism, obsessive compulsive behaviours and thinking, and difficulties in identifying and describing feelings. It is not clear to which disorder these symptoms should be attributed. Clinicians who do assessments might over diagnose or misdiagnose patients with ED and SUD as having PDs in this group given the overlap in symptoms between these disorders. Clinicians need to be cognizant that in the presence of other co-morbid disorders it can be difficult to accurately diagnose PD, particularly when patients have been struggling with concurrent SUD and ED problems for many years. Criteria which are used to diagnose PDs may be not applicable to cases with concurrent SUD and ED. It is important that clinicians evaluate what is the true source of shared symptoms.

There are stigmas associated with SUD, ED, and PD. These stigmas are perpetuated by the public, patients, and even mental health professionals [53–55]. Of these three disorders, PD may have the largest associated stigma. This is partially due to the chronic nature of personality problems, the difficulty of maintaining lasting personality change, and the fact that some of the behaviours in which these patients engage are perceived as difficult to treat [54–56]. Clinicians and patients can be frustrated by these treatment barriers. They can become increasingly negative towards the personality dysfunction and the patient’s resistance to change. Clinicians often expect that PD behaviours are inflexible, and some are unwilling to work with patients with a PD diagnosis [55,57]. It is important, therefore, that clinicians assess patients with ED and SUD carefully, so as not to mislabel symptoms of these disorders as personality traits.

The results of this pilot study and further research in this field could impact the way in which clinicians understand PD symptoms in patients with concurrent SUD and ED and could reduce the stigma of this population by clinicians who have viewed their patients’ apparent PD symptoms as difficult and untreatable. Altering the attitude of patients and clinicians may lead to greater optimism about change and therefore better treatment outcomes.

This study has limitations. The sample is small and involves only outpatients with concurrent SUD, ED, and PDs. The results might not be generalizeable and must be interpreted with caution as they raise questions that need answering in a larger and more detailed study. Nevertheless, the findings of this pilot study are exciting in that they have shown that treatment for ED and SUD may result in the remission of PD as well. They offer hope to clinicians and patients who are dealing with such concurrent disorders that change is possible, even for what may have been perceived as untreatable. They also may serve to remind clinicians to be cautious about diagnosing the existence of PD when SUD and ED have co-existed over long periods of time. Furthering this research may help revise existing theories of concurrent SUD and ED, and consequently assist clinicians in providing the highest level of care possible to patients with these challenging comorbid diagnoses.

## Figures and Tables

**Table 1.** Change in pre- to post-treatment mean scores on measures for Borderline, Avoidant, and Obsessive-Compulsive personality disorder symptoms.

Pre-treatment				Post-treatment				
Variable	n	Mean	S.D.	Mean	S.D.	t	df	p
SCL-90-R:								
Global Symptom Index High: >70 Moderate: 60 – 69 Average: 40 – 59 Below average: <40	20	69.78	7.53	58.78	11.10	4.59	19	0.000
Depression	20	70.10	4.19	60.75	10.12	4.06	19	0.001
Anxiety	18	65.61	10.58	59.89	9.18	2.58	17	0.010
Interpersonal Sensitivity	20	69.10	8.14	59.25	9.59	5.32	19	0.000
Psychoticism	20	68.20	8.17	59.40	8.33	4.36	19	0.000
Paranoid Ideation	20	62.05	10.80	52.10	9.11	5.67	19	0.000
Obsessive Compulsive	20	67.55	8.84	60.05	9.56	3.21	19	0.003
BDI-Total Score Minimal: <14 Mild: 14–19 Moderate: 20–28 Severe: >28	20	21.75	7.46	12.80	9.94	3.40	19	0.002
BAI-Total Score Minimal: <8 Mild: 8 – 15 Moderate: 16 – 25 Severe: >25	18	16.56	10.76	12.06	10.61	1.67	17	0.056
RAS:								
Over-Absorption Minimal: <17 Mild: 17–24 Moderate: 25–32 Severe: >32	20	24.00	7.98	19.35	7.60	2.99	19	0.004
EDI:								
Interpersonal Distrust Low: <2.3 Average: 2.3–5.1 Elevated: 5.2–8 Severe: >8.1	18	6.94	5.06	3.56	3.42	3.94	17	0.000
Perfectionism Low: <5.6 Average: 5.6–9.5 Elevated: 9.6–13.6 Severe: >13.7	18	9.33	5.42	7.44	4.68	2.34	17	0.016
Ineffectiveness Low: <2.1 Average: 2.1 Elevated: 5.7 Severe: >5.8	18	15.21	7.36	7.21	4.99	4.17	17	0.000
TAS:								
Total Score non-alexithymia: <52 possible alexithymia: 52–60 alexithymia: 61	13	57.15	12.72	48.23	9.26	2.53	12	0.026
Difficulty Identifying Feelings	13	21.54	6.58	17.23	5.99	1.86	12	0.088
Difficulty Describing Feelings	13	16.08	5.52	13.38	5.28	2.23	12	0.04

Note: SCL-90: Symptom Checklist-90-Revised; BDI: Beck Depression Inventory; BAI: Beck Anxiety Inventory; RAS: Reflective Activity Scale; TAS: Toronto Alexithymia Scale; EDI: Eating Disorder Inventory.

**Table 2.** Other symptom changes in pre- to post-treatment mean scores on treatment effectiveness measures for eating and substance use disorders.

Pre-treatment				Post-treatment				
Variable	N	Mean	S.D.	Mean	S.D.	t	df	p
Eating Disorder Examination								
Global Score Low: 0.127 Average: 0.932 Elevated:1.737 Severe: 2.452	16	3.74	0.98	1.52	1.04	5.96	15	0.000
Eating Disorder Inventory								
Bulimia Low: <1.8 Average: 1.8–4.8 Elevated: 4.9–7.9 Severe: >8.0	18	9.11	5.12	2.89	4.70	4.33	17	0.000
Drive for Thinness Low: <5.0 Average: 5.0–10.2 Elevated: 10.3–15.5 Severe: >15.6	18	12.11	5.75	6.50	5.24	3.40	17	0.002
Interceptive Awareness Low: <2.3 Average: 2.3–5.7 Elevated: 5.8–9.2 Severe: 9.3	18	11.56	5.85	4.72	6.94	3.19	17	0.003
Alcohol Severity Index Composite Score:								
Alcohol	15	0.36	0.32	0.20	0.26	2.67	14	0.009
Drug	14	0.15	0.16	0.08	0.08	1.42	13	0.002
Drug-Taking Confidence Questionnaire:								
Alcohol	11	56.59	29.10	72.95	27.29	−1.59	10	0.072
Drug	20	12.50	21.74	42.34	48.35	−3.43	19	0.001
